# Shrub density effects on the presence of an endangered lizard of the Carrizo Plain National Monument, California

**DOI:** 10.1002/ece3.10128

**Published:** 2023-05-19

**Authors:** Mario Zuliani, Nargol Ghazian, Malory Owen, Michael F. Westphal, H. Scott Butterfield, Christopher J. Lortie

**Affiliations:** ^1^ Department of Biological Science York University Toronto Ontario Canada; ^2^ US Bureau of Land Management, Central Coast Field Office Marina California USA; ^3^ The Nature Conservancy Sacramento California USA

**Keywords:** blunt‐nosed leopard lizard, density, desert, facilitation, foundation species, lizards, shrubs, telemetry

## Abstract

Positive associations between animals and foundational shrub species are frequent in desert ecosystems for shelter, resources, refuge, and other key ecological processes. Herein, we tested the impact of the density of the shrub species *Ephedra californica* on the presence and habitat use of the federally endangered lizard species, *Gambelia sila*. To do this, we used a 3‐year radio telemetry dataset and satellite‐based counts of shrub density across sites at the Carrizo Plain National Monument in San Luis Obispo County, CA. The effect of shrub density on lizard presence was contrasted with previous shrub cover analyses to determine whether measures of shrub density were superior to shrub cover in predicting lizard presence. Increasing shrub density increased lizard presence. As shrub density increased, lizards were located more frequently “above ground” versus “below ground” in burrows. Male lizards had significantly larger home ranges than females, but both sexes were similarly associated with increasing shrub densities. Shrub density and shrub cover models did not significantly differ in their prediction of lizard presence. These findings suggest that both habitat measures are effective analogs and that ecologically, both cover and the density of foundation shrub species are key factors for some desert lizards.

## Graphical abstract

Herein, we tested the influence of shrub density of the foundational shrub species *Ephedra californica* on the predicted resource use of the federally endangered blunt‐nosed leopard lizard *Gambelia sila* by joining a 3‐year telemetry dataset to ground‐truthed satellite imagery counts of all shrubs within a 20 m radius of each observed individual. As shrub density increased, we also found that lizards were located more frequently above ground versus below ground in burrows.

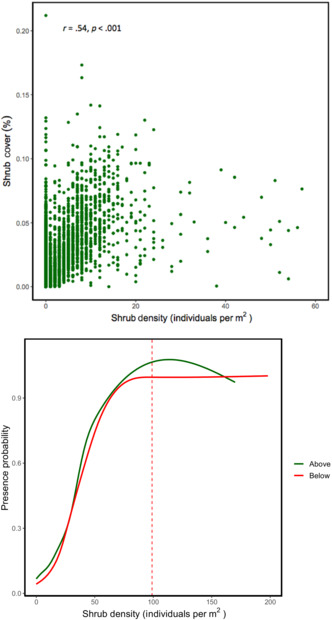

## 1 INTRODUCTION

Repopulation and protection of endangered species are driving factors for many restoration and conservation practices globally. For successful habitat protection and restoration, we must understand (1) species distribution within suitable habitat and (2) how that habitat is utilized—daily and seasonally (Elith et al., 2006; Eyre et al., 2022; Guisan & Thuiller, 2005). The relationship between animal species and their interactions with plants provides key insights into how managing plant communities can better support animal species (Lortie, Filazzola, et al., 2021; Zwolak et al., 2022). In arid/semi‐arid deserts and scrublands, animal species experience greater stress due to extremes in temperature and precipitation (Barrows, 2011; Van de Ven et al., 2020). Refuge from abiotic (Filazzola et al., 2018; Ivey et al., 2020) and biotic stressors primarily drive the dependency of organisms on ecosystem resources (Milchunas & Noy‐Meir, 2002; Nelson et al., 2007). Physiologically, reptilian species are highly dependent on thermoregulatory processes using exterior resources as they cannot maintain their internal temperature through metabolic heat (Ivey et al., 2020; Sunday et al., 2014). Thermoregulating reptile species such as lizards and snakes overtly exhibit this dependency, as extreme temperatures can only be avoided by seeking refuge above ground in/under vegetation or below ground in burrows, thus making these excellent species to study how resources are used within an ecosystem (Dematteis et al., 2022; Germano, 2019; Ivey et al., 2020; Sunday et al., 2014; Urban, 2015).

Significant research has been done on the endangered blunt‐nosed leopard lizard, *Gambelia sila*, including examining how this species uses the arid shrublands they inhabit (Gaudenti et al., 2021; Germano, 2019; Ivey et al., 2020; Lortie et al., 2020; Westphal et al., 2016; Westphal et al., 2018). This breadth of research has informed numerous conservation planning efforts (Bryant et al., 2020; Kelsey et al., 2018; Stewart et al., 2019) and has begun to inform land acquisitions and restoration efforts (Butterfield et al., 2021; Kelsey et al., 2018). *Gambelia sila* thermoregulates by basking in direct sunlight (Westphal et al., 2018; Ivey et al., 2020; Ivey et al., 2022; Gaudenti et al., 2021). *Gambelia sila* activity, “above ground” and “below ground,” changes as temperatures increase from spring to early summer (Germano et al., 1994). However, as ambient temperatures seasonally increase above certain thresholds (25–30°C) (Ivey et al., 2020), these individuals are forced to take refuge, either in underground burrows or in areas above ground with higher shrub cover (Germano, 2019; Ivey et al., 2020; Lortie et al., 2020). The home range for *G. sila* varies depending on the sex of the lizard but can range between 4 and 6 ha for males and 1 and 8 ha for females (Germano & Rathbun, 2016; Westphal et al., 2018). These home ranges are typically influenced by the presence and number of shrubs, suggesting that there may be an optimal number of shrub individuals that is sufficient for *G. sila* habitats (Westphal et al., 2018). With a higher number of shrubs within these areas, there is more opportunity for this species to thermoregulate as well as find food and shelter from predation (Westphal et al., 2016; Westphal et al., 2018). However, previous work by Germano and Rathbun (2016) suggests that while the home ranges of *G. sila* contain more shrub habitat, it is still possible for this species to travel and establish home ranges in the absence of shrubs. Challenges to thermoregulation—and dependency on the local environment for refuge—for lizards, including *G. sila*, are becoming more acute as global temperatures and the likelihood of drought events increase (Dell et al., 2014; Lortie et al., 2020; Sinervo et al., 2010; Westphal et al., 2016).

Lortie et al. (2020) found that there was a significant association between increasing shrub cover, the Normalized Difference Vegetation Index (NDVI), and *G. sila* presence—that is, an individual observation of each individual during data collection. To build off that work and to further examine the relationship between shrub density and lizard presence (Germano et al., 2011), we examined here whether shrub density could also be used, in addition to cover, to predict *G. sila* presence. Shrub density was selected for this study to further build on the finding previously reported by Zuliani et al. (2021, 2023) that shrub density influences the abundance and richness of local desert species while acting as an indicator of overall animal abundance. Here, we hope to extend these findings to a federally endangered species and show that shrub density is easier—and thus potentially cheaper and more cost effective for rapid range‐wide surveys—to measure, both in the field and with satellite‐based data.

While studies have evaluated the importance of shrub density on other vertebrate species within arid ecosystems (Zuliani et al., 2021, 2023), previous studies evaluating the importance of shrub density on *G. sila* presence relied on observational data (Westphal et al., 2018) and thus were not broadly applicable. To address these deficiencies and to increase the potential for an approach that could be more easily scaled across *G. sila*'s range, we developed methodology that uses radio telemetry and other *G. sila* presence data previously collected by Westphal et al. (2016) and Lortie et al. (2020) together with novel satellite‐based shrub density measurements to test the potential of free, easily accessible satellite data for predicting *G. sila* presence across large suitable landscapes. In this paper, we specifically examined the following questions:

1. Does *G. sila* presence increase with shrub density?
2. Do shrub density and cover similarly predict *G. sila* presence?
3. Is *G. sila* more likely to be observed above ground than below ground as shrub density increases?
4. Do home range and mean annual travel distance differ between male and female *G. sila*?

## 2 METHODS

### 2.1 Study species

*Ephedra californica* is the dominant shrub species within the Elkhorn Plain in the Carrizo Plain National Monument in San Luis Obispo County, CA, USA (35.11982, −119.62853) (Lortie, Filazzola, et al., 2021). *Ephedra californica* can reach heights of up to 1 m and typically takes 5–10 years to reach 0.5 m in size, suggesting that its cover and density changes over a longer period (Cutler, 1939; Filazzola et al., 2020; Lortie, Zuliani, et al., 2021). With numerous twigs and needle‐like leaves, this species possesses unique characteristics of both gymnosperms and angiosperms, making it well‐adapted to its native semi‐arid and arid environments (Loera et al., 2012). *Ephedra californica* is considered a foundational shrub species because it plays a disproportionately large role in structuring the system (Hawbecker, 1951; Westphal et al., 2018; Zuliani et al., 2021). This shrub species provides several unique resources to local lizard populations, including as a refuge from predators and as a place for thermoregulation (Braun et al., 2021; Gaudenti et al., 2021; Ivey et al., 2020; Lortie et al., 2018; Zuliani et al., 2021).

Within the Carrizo Plain National Monument, *G. sila* are found in both shrubbed and open areas in isolated populations (Gaudenti et al., 2021; Lortie et al., 2020; Westphal et al., 2018). Male *G. sila* individuals are territorial, minimally overlapping with other males (Germano, 2019; Westphal et al., 2018). *Gambelia sila* are mainly insectivores; however, they may consume smaller lizard species (Germano, 2019; Warrick et al., 1998; Westphal et al., 2018). The active season for *G. sila* adults lasts only ~3 months in the late spring to early summer, after which they transition into hibernation (Germano et al., 1994; Ivey et al., 2020). *Gambelia sila* is associated with shaded areas under shrub species and in underground burrows (Lortie et al., 2020), which they use as an additional way to thermoregulate (Gaudenti et al., 2021; Germano, 2019; Ivey et al., 2022). *Gambelia sila* will spend nights within burrows and may return to these burrows during the day when temperatures become too high (Warrick et al., 1998; Westphal et al., 2018).

### 2.2 Radio telemetry

We used *G. sila* location data previously collected using radio telemetry at the Carrizo Plain National Monument from 2016 to 2018 (Lortie et al., 2019, 2020; Noble et al., 2016; Westphal et al., 2018; Zuliani et al., 2021). *Gambelia sila* location data were collected on a total of 62 individuals, 36 males and 26 females. *Gambelia sila* individuals were tracked for 3 months (May–July) each year (2016–2018) during the time period of greatest activity for this species (Lortie et al., 2020; Westphal et al., 2018). Holohil model BD‐2 tags were attached to *G. sila* individuals using a small beaded chain, jewelry wire, and epoxy (Westphal et al., 2018). Collar weight (with the tag) ranged from 1.6 to 2.2 g, ensuring that the weight did not exceed between 5% and 10% of the lizard's body mass (Westphal et al., 2018). Each instance of *G. sila* presence was geolocated. Individual characteristics such as an individual's sex were also collected during the initial capture. Individuals located above ground and active were designated as “above ground,” while individuals judged to be below ground were designated as “below ground.” At each location, the presence of shrubs was recorded. *Gambelia sila* association with a shrub individual or in open areas was then recorded as microsite. Within the 3 years of observations, a total of 3553 *G. sila* observations were recorded with 1502 above ground and 2051 below ground (Lortie et al., 2020; Westphal et al., 2016). *Ephedra californica* is a long‐lived, slow‐growing shrub species and will thus not change in size and density over 3 years of continuous sampling (Bowers et al., 1995), allowing us to compare lizard presence and shrub density data from slightly different time periods. Additional details on the telemetry procedures can be found in Westphal et al. (2018).

Capture, collaring, and monitoring of *G. sila* were authorized by the United States Fish and Wildlife Service via permits TE166383‐4 (MFW), a Memorandum of Understanding issued to the California Department of Fish and Wildlife, and by the California Department of Fish and Wildlife via Scientific Collecting Permit SC‐2925 (MFW). The transportation, care, and use of lizards were in accordance with the Animal Welfare Act (7 U.S.C. 2131 et. seq.), which guides the US government use of vertebrates. We ameliorated any suffering of captured animals by allowing only trained personnel to handle them, and by limiting handling and housing time to the minimum necessary.

### 2.3 Shrub density data collection and ground truthing

*Ephedra californica* density data were derived from Google Earth using composite satellite imagery—digital images comprised of elements from several different images—from 2021. Composite imagery was developed using Landsat/Copernicus satellite data, with a 30 m spatial resolution. Satellite imagery was obtained for the same locations as the lizard telemetry tracking data within the Carrizo Plain National Monument, with an area of 6,613,399 m^2^. Each individual shrub located within the telemetry field site was geolocated and given a unique identification marker. Once all *E. californica* individuals were marked, Keyhole Markup Language files containing shrub locations were extracted and converted into a useable comma‐separated values file. Each shrub individual was given a unique latitude and longitude value corresponding to their location within the field site. We used R version 4.2.1 (R Core Team, 2022) to determine the density of *E. californica* individuals within a 20 m radius of each *G. sila* presence. A total of 200 random shrub locations were then selected and ground‐truthed in the field to determine the accuracy of our satellite imagery‐based shrub density measurements. In situ shrub density was determined by locating each of the randomly selected 200 shrubs and measuring the number of shrubs within a 20 m radius. Shrub cover, measured as percent cover within a 20 m radius, was extracted from shrub cover data previously collected via Landsat 8 by Lortie et al. (2020). We also resampled NDVI data at a 20 m radius, similar to what Lortie et al. (2020) did with their investigation of shrub cover and lizard presence. NDVI was calculated as NDVI = (NIR – RED)/(NIR+RED) (Zaitunah et al., 2018) and was included here as it is known to be a strong predictor of vegetation greenness and primary production (Butterfield & Malmström., 2009; Ju & Masek, 2016) and is therefore commonly used to quickly identify vegetated areas (Zaitunah et al., 2018). NDVI and shrub density were then both used to determine whether these factors influenced *G. sila* presence. All data are publicly published and available on the Knowledge Network of Biodiversity (Zuliani et al., 2022).

## 3 STATISTICAL ANALYSIS

All statistics and models were done in R 4.2.1 (R Core Team, 2022). The *ResourceSelection* R package (Lele et al., 2019) was used to model *G. sila* shrub use based on density and cover (Lortie et al., 2020). The distinct function was used in base R 4.2.1 to remove any duplicates within the lizard presence data. Data were filtered and compared based on similarity of latitude, longitude, lizard identification, year, and microsite. Individuals with identical latitude and longitude coordinates within the same year were removed to minimize the probability of duplication. In addition, individuals who were located at the same latitude and longitude associating with the same microsite were also filtered. We also tested the effects of rounding the latitude and longitude to 4 and 3 decimal points on lizard duplicate removal. Rounding the lizard geolocations to 4 decimal places reduces the distance of each coordinate by 11 m while rounding the geolocations to 3 decimal places reduces the distance of each coordinate by 111 m. This was conducted in a variety of combinations and compared with the raw data. The SF package was used to calculate the mean annual distance traveled by individual lizards using three different duplication removal methods (Table S1; Pebesma, 2018). These methods were selected as they provided the most meaningful data after filtering for possible duplicates. 1000 pseudo‐absence data points were then generated using the dismo R package to simulate areas where *G. sila* individuals were not located (Hijmans et al., 2022). The resource selection probability function (rspf) was used to estimate the probability of a lizard individual in shrubbed areas (Lele et al., 2013; Roberts et al., 2017). In addition, sex and year were used as a factor with shrub density to estimate the resource selection of shrub density between male and female individuals across 3 years of telemetry data. This function estimates the frequency of occurrence of a species for a specific factor such as shrub density and cover (Roberts et al., 2017). Akaike information criterion (AIC) scores were generated for both shrub density and shrub cover models and compared as both strongly correlated with lizard shrub use (Lele et al., 2013; Lortie et al., 2020; Roberts et al., 2017). The scores determined which model best suited the data—based on a lower AIC score. Pearson's Product–Moment Correlation was used to determine the strength and directions of the relationship between shrub density and cover. An approximation of second derivative for the data points for the mean fitted use by shrub density curve was used to determine the inflection point of fit (Christopoulos, 2014). Maximum likelihood estimates were used with shrub density, shrub cover, and ground use (above vs. below) as predictor variables. Shrub density and cover were analyzed against lizard presence to depict their relationship. Home range sizes were then calculated using 95% and 100% minimum convex polygon areas, using the adehabitatHR package (Calenge, 2006; Mohr, 1947). Both male and female *G. sila* home ranges were calculated and compared across the 3 years of telemetry data. General linear models were then used to compare shrub density and shrub cover use by both male and female *G. sila* individuals.

## 4 RESULTS

**FIGURE 1:**
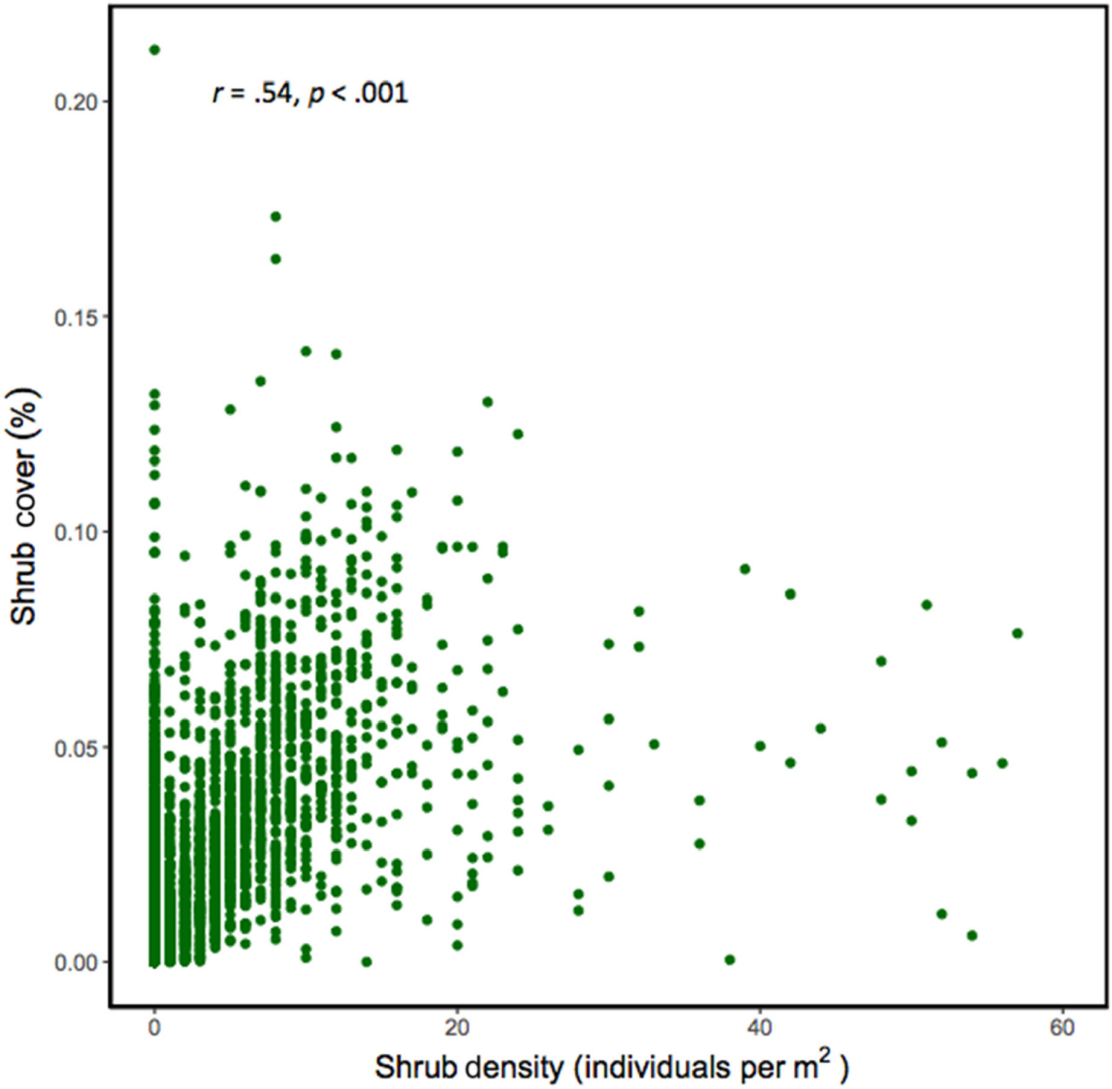
Relationship between shrub density (number of *Ephedra californica* individuals per 20 m^2^ radius) and shrub cover for *E. californica* within the Carrizo Plain National Monument study site. Total shrub density was joined with estimate shrub cover from imagery data to test for a correlation (*r* = .54, *p*‐value < .001).

**FIGURE 2:**
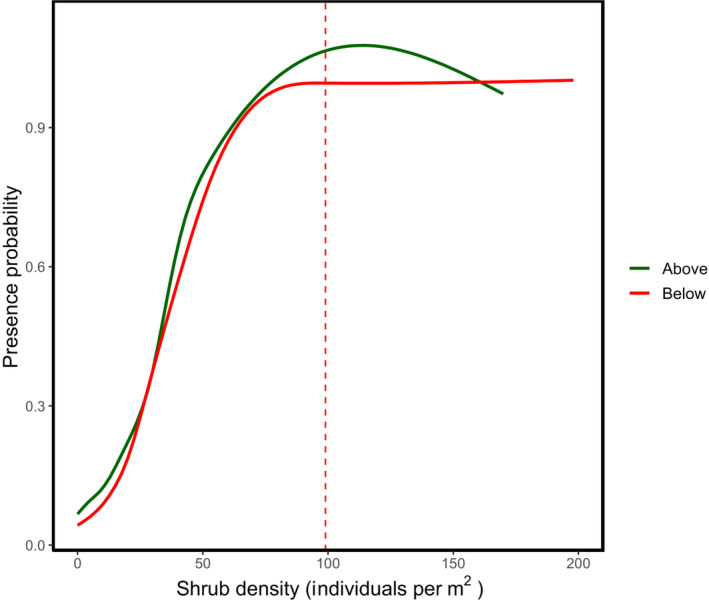
Relative effects of shrub density (number of *Ephedra californica* individuals per 20 m^2^ radius) on *Gambelia sila* presence probability above and “below ground” for each individual presence. Radio telemetry data were joined with geolocated shrub data to estimate the shrub density within a 20 m radius of each *Gambelia* observation. The data were then grouped by above and “below ground.” Shaded areas show a 95% confidence interval band for the lines of best fit.

**FIGURE 3:**
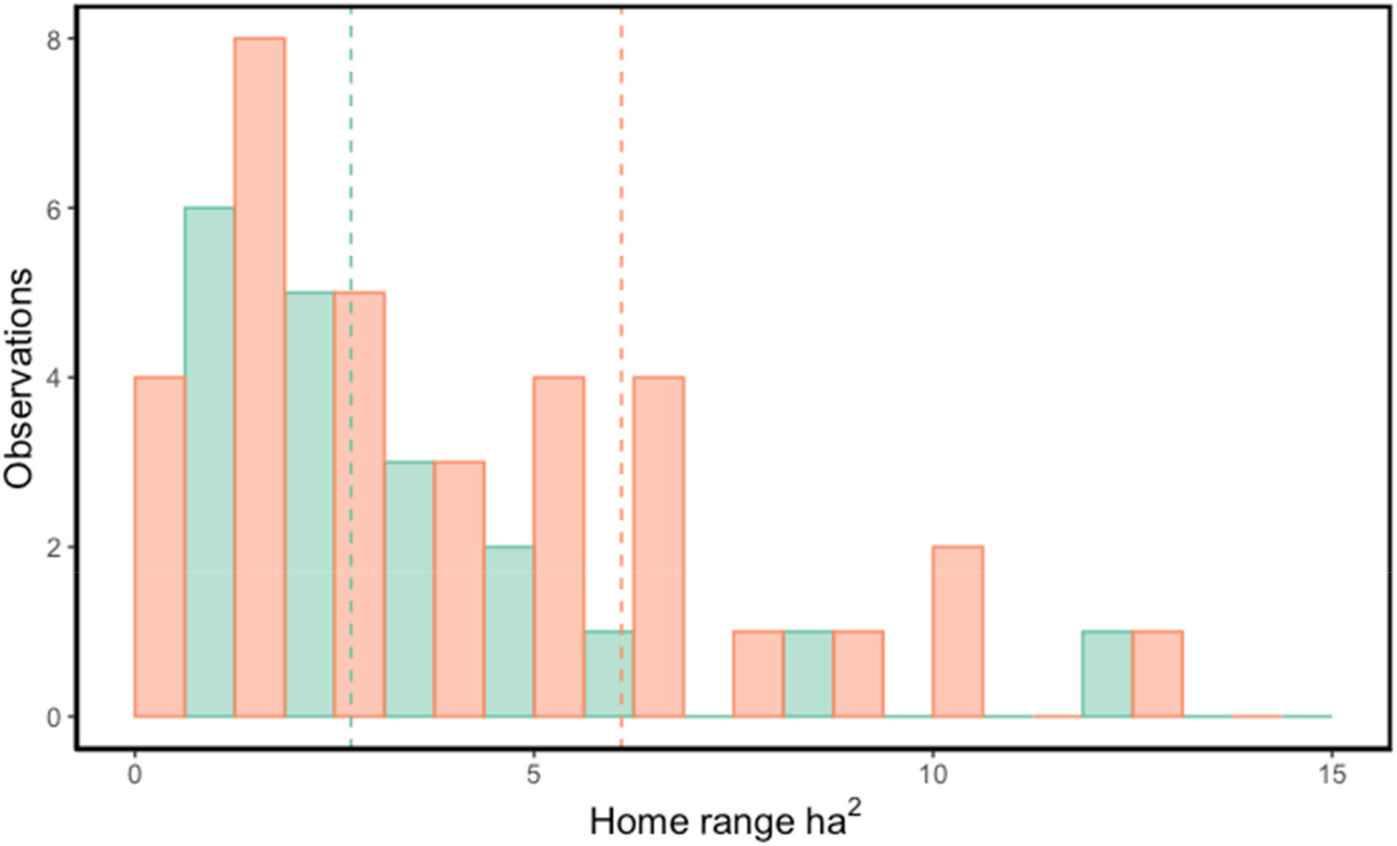
Relative home range of *Gambelia sila* individuals (ha^2^) across 3 years of radio telemetry tracking. Home range sizes were calculated using the adehabitatHR package in R 4.2.1. Individuals were separated based on lizard sex with blue representing females and red representing males. Dashed lines represent the average home range of male and female individuals independently.

There were no statistically significant differences between the geolocated shrub density estimates and the ground‐truthed shrub density counts in the field (Paired *t*‐test, *t* = −.048, df = 389.41, *p* = .962). Shrub cover and density were positively correlated (Figure 1, Pearson's Product–Moment Correlation, estimate = 0.369, *t*‐value = 23.761, df = 3571, *p* < .001). The AIC score for the shrub density rsf model was lower than the shrub cover model, suggesting that density is a more parsimonious model fit for presence data (Snipes & Taylor, 2014) (Table S2; AIC scores density = 11,253.37, cover = 11,257.06). Shrub density significantly predicted the presence of *G. sila*, for both the above ground and below ground categories (Figure 2, AIC = 11,289.36, estimate = 0.088 ± 0.006, *p* < .001). The inflection point of the predicted use curve for lizards by shrub density was 99 shrubs per 20 m radius plot (or about 100 shrubs per 20 m^2^) (Figure 2). Lizard presence was significantly greater above ground at higher shrub densities (Figure 2, rspf estimate = −0.380  ±0.083, *p* < .001). The removal of *G. sila* presence duplicates had no impact on model outcomes (Table S3). NDVI positively correlated with lizard presence (Table S3). Mean annual distance traveled by *G. sila* individuals was significant across all duplicate removal methods (Table S1; *t* = 19.026, df = 215, *p* < .001). Mean annual distance traveled was significantly different across all duplicate removal methods except between methods 2 and 3 (Tukey, estimate = −9.8, *t*‐value = −1.457, *p* = .3139). Shrub density significantly predicted the presence of male (rspf estimate = 0.06851 ± 0.0118, *p* < .001) and female *G. sila* individuals (rspf estimate = 0.06538 ± 0.0071, *p* < .001). Male *G. sila* had significantly greater home range sizes in 2018 sampling (Figure 3; Table S4; *t*‐test = −2.318. df = 56, *p* = .0241).

## 5 DISCUSSION

The major finding of this study is that the presence of *G. sila* individuals increased with higher shrub density, up to a plateau, which we identified was 100 shrubs per 20 m^2^, or 25% shrub cover (Lortie et al., 2020). Higher shrub densities were also associated with increased likelihood of lizard presence “above ground” versus “below ground.” Measurements of NDVI in our shrub density models positively predicted the presence of *G. sila* individuals, similar to conclusions drawn from previous work by Lortie et al. (2020) showed that both shrub cover and NDVI positively predicted lizard presence. Male lizards had larger home ranges than female individuals for the 2018 sampling season only. Males were frequently associated with higher density shrub areas, but the predicted habitat use was not significantly different between lizard sexes, suggesting that shrub density and cover are important as potential resources for both male and female *G. sila* (Lortie et al., 2020; Ivey et al., 2022). Increasing shrub densities increases the likelihood that a lizard will encounter and interact with a shrub (Zuliani et al., 2021), providing stopping points or distributed refuges for individuals as they move within their home range.

Landscape features and various natural resources are important factors to consider in ecological surveys that observe associations. Typically, in arid/semi‐arid ecosystems, shrub species provide benefits to local animal communities through buffering climatic extremes, acting as refuges from potential predation, and other abiotic and biotic conditions (Bruno et al., 2003; Eldridge & Soliveres, 2014; Filazzola et al., 2017; Ivey et al., 2022; Noble et al., 2016; Westphal et al., 2018). Areas with higher shrub cover allow for lower thermal amplitudes, while areas with higher density can consist of smaller shrubs utilized for abiotic stress amelioration, which provides multiple areas for individual utilization (Hollzapfel & Mahall, 1999; Filazzola et al., 2017). This behavior extends beyond *G. sila* as several other reptile species utilize shrub cover and density to ameliorate abiotic stressors. For instance, *Psammodromus algirus*, the Algerian sand racer, utilizes shrubs within the Tajo Basin of Spain (Diaz & Carrascal, 1991; Zamora‐Camacho et al., 2016) for thermoregulation, predator avoidance, and movement minimization (Diaz & Carrascal, 1991). These facilitative associations, in which one species benefits while the other is unaffected (Molina‐Montenegro et al., 2016), can be subdivided into facultative (being able to live with the presence or absence of an environmental condition), and obligate, where one species requires the benefits from another to exist (Butterfield, 2009). The relationship observed in our study is more facultative than obligate, as *G. sila* will utilize shrubs when present; however, if absent, individuals can still be present within a site. With more shrubs, there are likely more opportunities for positive associations as even relatively small shrub individuals provide benefits (Gaudenti et al., 2021; Lortie et al., 2018; Zuliani et al., 2021).

Shrubs can provide refuge for lizards to thermoregulate and escape harsh abiotic conditions, so that they might stay above ground even when below ground temperatures are more optimal (Gaudenti et al., 2021). Shrub cover and density therefore potentially provide crucial opportunities for lizard thermoregulation and can be utilized to predict the frequency of individual lizard observations (Filazzola et al., 2017; Lortie et al., 2020; Zuliani et al., 2021). This contrasts with lizard individuals in open or relatively shrub‐free habitats, which are more reliant on burrows to reduce their body temperature, similar to other desert species (Bean et al., 2014; Gaudenti et al., 2021; Germano & Rathbun, 2016; Ivey et al., 2020). Males also generally extended their home ranges in this ecosystem, suggesting that refuge provided by foundational plant species can be important stopping or foraging points (Ivey et al., 2020, 2022). While female *G. sila* individuals similarly utilize these areas for foraging points, a large portion of their active season is spent underground while breeding (Germano, 2019; Ivey et al., 2020). The influence of shrub density on the thermoregulation, behavior, and associations of blunt‐nosed leopard lizards with habitat further illustrates how these resources are beneficial to this endangered species and how important relevant associations are to their conservation and restoration.

## 6 CONCLUSION

Shrub density can be utilized to predict the presence of *G. sila* individuals within arid California ecosystems. This can guide species conservation and restoration efforts through both the preservation of shrubbed—and likely suitable and occupied habitat—landscapes and the restoration of areas that could be suitable if shrubs were planted. Shrub density was positively correlated with shrub cover suggesting that both are viable measures that can be used to predict lizard presence. However, we suggest measures of shrub density are easier to collect in the field and are thus easier and cheaper to collect across large areas. Restoring areas with *E. californica* or preserving already established areas can benefit *G. sila* populations as these areas can act as distributed refuges for these individuals. There is a significant current opportunity to retire and restore agricultural land within the San Joaquin Valley in Southern California (Bryant et al., 2020; Butterfield et al., 2021; Kelsey et al., 2018). Restoring these ecosystems could lead to *G. sila*—as well as more than 20 other threatened and endangered species—recovery. Our findings can help guide these restoration efforts, identifying specific density and cover thresholds that will provide the best potential habitat for *G. sila* individuals.

## AUTHOR CONTRIBUTIONS

**Mario Zuliani:** Conceptualization (equal); data curation (equal); formal analysis (equal); investigation (equal); methodology (equal); project administration (equal); visualization (equal); writing – original draft (lead); writing – review and editing (lead). **Nargol Ghazian:** Data curation (equal); formal analysis (equal); writing – review and editing (equal). **Malory Owen:** Writing – review and editing (supporting). **Christopher J. Lortie:** Conceptualization (equal); data curation (equal); formal analysis (equal); funding acquisition (equal); investigation (equal); methodology (equal); project administration (equal); supervision (equal); validation (equal); visualization (equal); writing – original draft (equal); writing – review and editing (equal). **Michael F. Westphal:** Conceptualization (equal); methodology (equal); writing – review and editing (equal). **H. Scott Butterfield:** Conceptualization (equal); methodology (equal); writing – review and editing (equal).

## FUNDING INFORMATION

This research was made possible through the Natural Sciences and Engineering Research Council of Canada (NSERC NG) grant awarded to CJL and the York University Faculty of Graduate Studies (FGS) award granted to Mario Zuliani. The BLM and TNC supported field data collection.

## Supporting information


Table S1:
Table S2:
Table S3:
Table S4:

## Data Availability

All data are freely available at figshare (DOI: https://doi.org/10.6084/m9.figshare.8239667.v2).
