# Supplementary material for: Shrub density effects on the presence of an endangered lizard of the Carrizo Plain National Monument, California
Source: Ecol Evol. 2023 May 19;13(5):e10128. doi: 10.1002/ece3.10128 (PMC10199236; doi:10.1002/ece3.10128)
Supplement: Supplementary file 1 — Table S1: Table S2: Table S3: Table S4: [file ECE3-13-e10128-s001.docx]

**Supplementary Appendix:**

**Table 1A:** Mean annual traveled distance (m) by *G. sila* individuals across 3 years within the Carrizo Plain National Monument, after duplicate removal. Data was calculated using the sf package in R 4.2.1.

| method | dataframe | year | mean annual distance (m) | standard error |
| --- | --- | --- | --- | --- |
| 1 | distinct() | 2016 | 22.55 | 2.63 |
|  |  | 2017 | 31.88 | 4.61 |
|  |  | 2018 | 33.84 | 3.82 |
| 2 | distinct(year, lizard, lat, long) | 2016 | 74.84 | 11.15 |
|  |  | 2017 | 58.44 | 4.91 |
|  |  | 2018 | 99.65 | 9.84 |
| 3 | distinct(year, lat, long, microsite) | 2016 | 59.05 | 7.23 |
|  |  | 2017 | 56.48 | 5.67 |
|  |  | 2018 | 90.18 | 10.44 |

**Table 2A:** Akaike’s information criterion (AIC) and related measures used for model selection of variables, based on the resource selection probability function (rspf). Models comparing shrub cover and density were used to determine which can be used to predict *G. sila* presence.

| **Model** | **k** | **AIC** | **∆AIC** | **ω** |
| --- | --- | --- | --- | --- |
| Density | 7 | 11253.37 | 0.00 | 0.864 |
| Cover | 7 | 11257.06 | 3.69 | 0.136 |

**Table 3A:** The effects of duplicate removal on *G. sila* presence and rspf estimates. Data was filtered using the distinct() function in base R 4.2.1. Data were filtered based on unique values across key factors including; latitude, longitude, lizard identification, year, and microsite.

| dataframe | n | predictor | estimate | std.error | z value | p-value |
| --- | --- | --- | --- | --- | --- | --- |
| all relocations | 9307 | density | 0.107967 | 0.002296 | 47.033 | **< 0.001** |
|  |  | NDVI | 8.474163 | 0.784914 | 10.796 | **< 0.001** |
|  |  | ground | 0.041556 | 0.032654 | 1.273 | 0.203 |
| distinct() | 8006 | density | 0.112317 | 0.002496 | 44.992 | **< 0.001** |
|  |  | NDVI | 11.24338 | 0.826968 | 13.596 | **< 0.001** |
|  |  | ground | 0.073399 | 0.037035 | 1.982 | 0.0475 |
| distinct(year, lizard, lat, long) | 4690 | density | 0.082796 | 0.005537 | 14.952 | **< 0.001** |
|  |  | NDVI | 13.892995 | 1.441683 | 9.637 | **< 0.001** |
|  |  | ground | -0.627815 | 0.072326 | -8.68 | **< 0.001** |
| Rounded 4 decimal (within ~11m) | 4690 | density | 0.082796 | 0.006648 | 12.455 | **< 0.001** |
|  |  | NDVI | 13.892995 | 1.539875 | 9.022 | **< 0.001** |
|  |  | ground | 0.627815 | 0.062725 | -10.009 | **< 0.001** |
| Rounded 3 decimal (within ~111m) | 3532 | density | 0.085484 | 0.008311 | 10.286 | **< 0.001** |
|  |  | NDVI | 11.023229 | 2.841259 | 3.88 | **< 0.001** |
|  |  | ground | -0.546276 | 0.117606 | -4.645 | **< 0.001** |
| distinct(year, lat, long, microsite) | 4508 | density | 0.087971 | 0.005717 | 15.388 | **< 0.001** |
|  |  | NDVI | 13.31861 | 1.605133 | 8.298 | **< 0.001** |
|  |  | ground | -0.64207 | 0.06684 | -9.794 | **< 0.001** |

**Table 4A:** The minimum convex polygon (MCP) area estimates for male and female *G. sila* individuals across 3 years of radio telemetry tracking at the Carrizo Plain National Monument. All individuals with 5 or more presences were included. Estimates were compiled using the adehabitatHR package in R 4.2.1.

| **Year** | **Sex** | **n** | **Home Range (ha)** | **Standard Error** |
| --- | --- | --- | --- | --- |
| 2016 | F | 10 | 1.70 | 0.458 |
| 2016 | M | 11 | 4.76 | 2.047 |
| 2017 | F | 5 | 1.08 | 0.297 |
| 2017 | M | 10 | 1.82 | 0.406 |
| 2018 | F | 11 | 4.36 | 1.082 |
| 2018 | M | 15 | 9.93 | 2.601 |
